# A high‐quality haplotype‐resolved genome of *Pyrus calleryana* provides insights into adaptive gene families

**DOI:** 10.1002/tpg2.70286

**Published:** 2026-07-29

**Authors:** Xiaogang Dai, Xiaoyue Yang, Changci Jiang, Lizhen Huang, Fangping Li, Zhuangwei Hou, Fenghou Shi, Zefu Wang, Wenjie Yang, Yongbao Shen

**Affiliations:** ^1^ State Key Laboratory of Tree Genetics and Breeding, Co‐Innovation Center for Sustainable Forestry in Southern China, College of Forestry and Grassland Nanjing Forestry University Nanjing China; ^2^ College of Ecology and Environment Nanjing Forestry University Nanjing China; ^3^ Guangdong Provincial Key Laboratory of Plant Molecular Breeding Guangdong Basic Research Center of Excellence for Precise Breeding of Future Crops South China Agricultural University Guangzhou China; ^4^ Shenzhen Branch, Guangdong Laboratory for Lingnan Modern Agriculture, Genome Analysis Laboratory of the Ministry of Agriculture, Agricultural Genomics Institute at Shenzhen Chinese Academy of Agricultural Sciences Shenzhen China

## Abstract

Callery pear (*Pyrus calleryana*) is a deciduous species native to East Asia with notable ornamental and ecological value, but the genomic resources for this species remain limited. Here, we report the first haplotype‐resolved, chromosome‐scale genome of *P. calleryana*. The final genome assembly comprises two phased haplotypes measuring 506.97 and 504.62 Mb. These haplotypes contain 41,234 and 41,329 predicted protein‐coding genes, respectively, with more than 97% of the genes receiving functional annotations. Comparative genomic analyses further identified 680 species‐specific orthogroups enriched in hormone signaling, metabolic processes, and stress‐responsive pathways, among which several LIK1‐like genes may contribute to environmental adaptation. This high‐quality genome assembly offers an essential foundation for subsequent studies on the ecological adaptability, invasiveness, and functional genomics of *P. calleryana*.

## Plain Language Summary

Callery pear is a useful East Asian tree with ornamental and breeding value, but it lacks high‐quality genome data. We aimed to build its first chromosome‐level, haplotype‐resolved genome to study its adaptation and evolution. We assembled two high‐quality haplotypes, annotated over 41,000 genes each, and found 680 unique orthogroups linked to stress resistance. This genome provides a key resource for understanding its adaptability, invasiveness, and supporting pear breeding and ecological research.

**Abbreviations**
LAI: LTR Assembly Index
LTR: long terminal repeat
SNP: single nucleotide polymorphism
TEs: transposable elements
TIRs: terminal inverted repeats

## 1 INTRODUCTION

**FIGURE 1:**
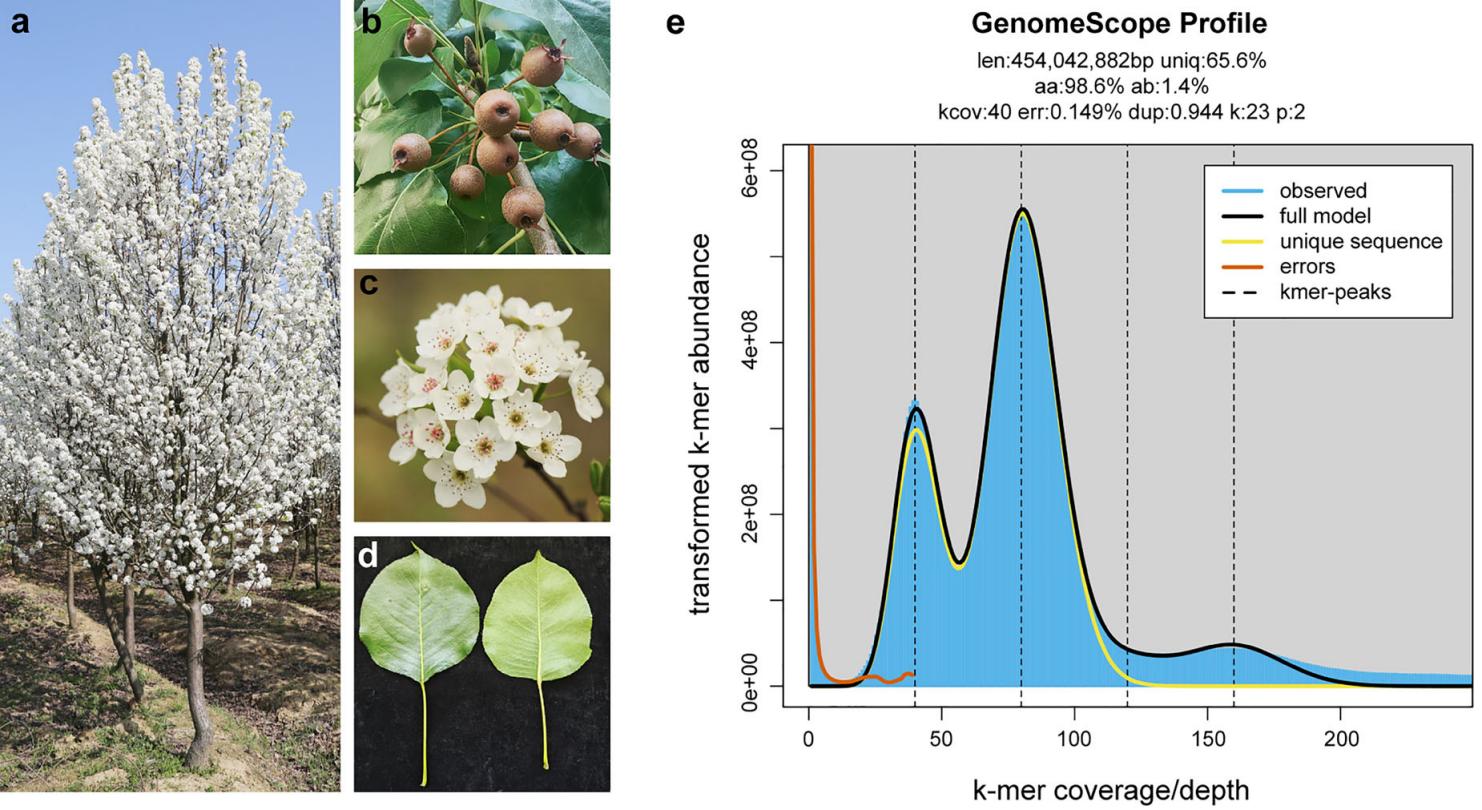
Plant morphology and genome size estimation of *P. calleryana*: (a) mature plant, (b) flower, (c) fruit, (d) leaf, and (e) 23‐kmer distribution estimation. The x‐axis indicates k‐mer coverage/depth, and the y‐axis indicates transformed k‐mer abundance, calculated as coverage × k‐mer frequency. With kcov = 40 and ploidy = 2, the peak near 40× corresponds mainly to heterozygous single‐copy k‐mers, whereas the major peak near 80× corresponds to homozygous unique k‐mers. The low‐coverage component represents putative sequencing errors, and higher coverage peaks or shoulders are associated with repetitive or duplicated k‐mers.

Callery pear (*Pyrus calleryana* Decne.) is a deciduous member of the Rosaceae, originating from East Asia and naturally distributed across China, Korea, and Japan (Bell & Zimmerman, 1990; Sapkota et al., 2021). It grows widely in diverse habitats ranging from lowlands to mountain slopes and exhibits strong adaptability to different soil and climatic conditions (Nowicki et al., 2022; Sapkota et al., 2021). The species is characterized by dense white inflorescences that bloom in early spring, glossy green foliage, and small, hard fruits (Figure 1a‐d). Owing to its high ornamental value and environmental tolerance, *P. calleryana* has been widely planted in urban landscapes and is also used as a rootstock for other *Pyrus* cultivars (Robbani et al., 2006). Because of its high adaptability and extensive cultivation, the species has also become naturalized and invasive in parts of North America (Culley & Hardiman, 2007). Meanwhile, *P. calleryana* readily hybridizes with cultivated pear species and has played an important role in pear breeding and germplasm innovation (Culley & Hardiman, 2009). Its broad ecological tolerance, high genetic diversity, and close relationship with cultivated pears also make it a useful species for investigating adaptation and evolution within the genus *Pyrus*.

Although *P. calleryana* holds considerable ecological, horticultural, and genetic value, available genomic resources for this species are still limited. Several genome assemblies of *Pyrus* species have been released in recent years, including those of cultivated pears and a few wild relatives (Cao et al., 2025; Dong et al., 2020; Gao et al., 2021; Linsmith et al., 2019; M. Sun et al., 2023, 2025; Wu et al., 2013; X. Zhang et al., 2025). However, a high‐quality, chromosome‐scale genome assembly for *P. calleryana* has not been reported. Most research on *P. calleryana* has focused on transcriptomic studies, such as investigating genes in response to salt stress (Matsumoto et al., 2006; Y. Xu et al., 2015) and transcriptomic responses to bacterial canker (Kan et al., 2017). Additionally, the complete chloroplast genome of *P. calleryana* has been sequenced, providing valuable data for phylogenetic studies (Nowicki et al., 2022). However, the absence of a reference genome has limited comprehensive investigations into its functional genomics and adaptive strategies.

In this study, we generate the first chromosome‐scale, haplotype‐resolved genome assembly of *P. calleryana* using an integrated strategy that combines Pacific Biosciences (PacBio) High Fidelity (HiFi) long reads with Hi‐C scaffolding. The assembly was organized into two sets of 17 pseudochromosomes, which is consistent with its previously reported chromosome number (Nowicki et al., 2022; Sapkota et al., 2021). This chromosome‐level phased assembly establishes an essential genomic framework for investigating the ecological adaptation, invasive potential, and functional traits of *P. calleryana*, thereby supporting future research efforts on this species.

## 2 MATERIALS AND METHODS

### 2.1 Plant materials and genomic sequencing

Samples of *Pyrus calleryana* were collected from a single naturally growing individual located at Baima Campus of Nanjing Forestry University (31°36′28.92″ N, 119°10′46.46″ E). The sampling of *P. calleryana* involved non‐protected plant material collected from publicly accessible locations. According to local regulations, no specific permits or approvals were required for sample collection. Young leaves were frozen in liquid nitrogen immediately after sampling and stored at −80°C.

Genomic DNA was extracted using a cetyltrimethylammonium bromide (CTAB)‐based protocol and subsequently purified and size‐selected with AMPure PB beads (Doyle, 1987). A SMRTbell library was generated and sequenced on the PacBio Revio platform to obtain HiFi reads. For Hi‐C sequencing, fresh leaves were fixed with formaldehyde, processed with DpnII following established Hi‐C methods (van Berkum et al., 2010), and the final library was sequenced on the DNBSEQ‐T7 platform (BGI).

### 2.2 Genome assembly

Genome size and heterozygosity were first estimated from 23‐mer frequency distributions using KMC (Kokot et al., 2017) and GenomeScope 2.0 (Ranallo‐Benavidez et al., 2020). The assembly process integrated HiFi long reads and Hi‐C short reads using Hifiasm v0.24.0 (Cheng et al., 2021) under the Hi‐C integrated assembly mode with other parameters set to default, enabling the generation of contigs representing two distinct haplotypes. After that, contamination screening was subsequently conducted on the contig‐level assemblies using Tiara v1.0.3 using default parameters (Karlicki et al., 2022), and contigs classified as potential contaminants were excluded from downstream analyses. The two contig assemblies were anchored to chromosomes using Hi‐C data through HapHiC v1.0.6 (Zeng et al., 2024). The resulting assemblies were then manually curated using Juicebox v1.11.08 (Durand et al., 2016) to correct misjoins and refine contig orientations. This step ensured accurate chromosomal architecture and continuity. Final phasing was achieved based on Hifiasm outputs, resulting in the separation of the two haplotypes, designated as Haplotype A and Haplotype B. Additionally, the telomeric sequences were identified using quarTeT (Lin et al., 2023), and centromeric regions were subsequently detected across all chromosomes using CentIER v2.0 (D. Xu et al., 2024); both tools were run with default parameters. We employed three complementary approaches to evaluate the reliability of this final genome assembly: Benchmarking Universal Single‐Copy Orthologs (BUSCO) with eudicotyledons_odb12 dataset in genome mode (Seppey et al., 2019), the LTR assembly index (LAI), and the consensus quality value (QV) (Rhie et al., 2020).

### 2.3 Repeat annotation

De novo identification and annotation of transposable elements (TEs) were carried out using EDTA pipeline v2.2.2 with default parameters (Ou et al., 2019). This pipeline enabled the comprehensive classification of TEs into two major classes: Class I retrotransposons and Class II DNA transposons. Major subclasses, including long terminal repeat (LTR) retrotransposons, terminal inverted repeat (TIR) DNA transposons, and Helitrons, were further identified and summarized.

### 2.4 Gene structure prediction and functional annotations

Structure prediction was conducted using a combination of ab initio and evidence‐based strategies. For ab initio predictions, ANNEVO v2.0 (https://github.com/xjtu‐omics/ANNEVO) was run with the parameter “–lineage Embryophyta,” and Helixer v0.3.4 (Holst et al., 2023) was run with the parameter “–lineage land_plant” to identify coding regions. To further enhance model accuracy, EviANN v2.0.2 (Zimin et al., 2025) was employed to integrate transcriptome evidence, thereby refining gene boundaries and exon–intron structures. Transcriptomic datasets were retrieved from NCBI (Kan et al., 2017; J. Zhang, Qian, et al., 2022) (Table S1) and aligned to the genome to support annotation refinement. Annotation quality was evaluated at both structural and completeness levels. Structural features of the predicted gene models were quantified using summary statistics, including the numbers of genes, CDSs, mRNAs, and exons, as well as the distributions of transcript length, exon number per transcript, and exon length. Annotation completeness was further evaluated using BUSCO analysis on the predicted proteome.

Functional annotation of protein‐coding genes was carried out using several complementary strategies. First, KEGG pathway information was assigned using the KEGG Automatic Annotation Server (https://www.genome.jp/tools/kaas/). Then, gene sequences were annotated against the eggNOG 5.0.2 database with eggNOG‐mapper v2.1.12 (Cantalapiedra et al., 2021). To further characterize gene functions, similarity searches were performed with BLAST 2.14.1+ (Camacho et al., 2009) against both the Swiss‐Prot and TrEMBL protein databases with an *E*‐value cutoff of 1e−5. Gene Ontology (GO) terms were then obtained using Blast2GO v2.5 (Conesa et al., 2005). The resulting annotations from all sources were integrated to generate comprehensive functional descriptions for each gene.

### 2.5 Comparison between haplotype assemblies

To evaluate collinearity between the two haplotype assemblies, inter‐haplotype synteny was analyzed using JCVI (Tang et al., 2024). Protein‐coding gene annotations and genome coordinates from Haplotype A and Haplotype B were converted into the required input formats, and collinear gene anchors between the two haplotypes were identified using the JCVI pipeline with parameters “–no_strip_names –cscore = .99.” Only syntenic blocks connecting Haplotype A and Haplotype B were retained, whereas within‐haplotype self‐comparisons were not included. The resulting inter‐haplotype collinear blocks were visualized using Circos (Krzywinski et al., 2009). Links with endpoint intervals shorter than 10 bp were not displayed during visualization. To compare the two haplotypes of *P. calleryana* assembly, we applied SyRI v1.6.3 (Goel et al., 2019) to detect structural variations ≥50 bp, as well as single nucleotide polymorphisms (SNPs) and small InDels (< 50 bp). The resulting syntenic blocks and major genomic rearrangements were displayed using Plotsr v1.1.1 (Goel & Schneeberger, 2022).

### 2.6 Gene family identification and phylogenetic analysis

To infer phylogenetic relationships, *Pyrus bretschneideri*, *Pyrus communis*, *Pyrus pyrifolia*, *Pyrus betulifolia*, *Malus domestica*, and *Prunus persica* were selected for comparative analysis. *Arabidopsis thaliana* was included as an outgroup species. All genome assembly and gene annotation datasets were retrieved from public databases (Table S2). Orthologous gene families across the selected species were identified using OrthoFinder v2.5.5 with default parameters (Emms & Kelly, 2019). This tool groups homologous genes into orthogroups based on sequence similarity. From these orthogroups, single‐copy genes present in all species were extracted for phylogenetic analysis. Each single‐copy ortholog was independently aligned with MAFFT v7.526 using the –auto option (Katoh & Standley, 2013), and the resulting alignments were concatenated to generate a supermatrix to construct a maximum‐likelihood phylogeny in RAxML v1.2.2 (Stamatakis, 2014) using the JTT+I+G+F substitution model with 100 bootstrap replicates.

To calibrate the molecular clock, we used the divergence time between *A. thaliana* and *P. communis* obtained from TimeTree (http://www.timetree.org), which is approximately 108 Mya (102–113 Mya). This calibration point was subsequently applied in MCMCTree (Yang, 2007) to infer the divergence times among lineages under a Bayesian relaxed‐clock framework. We analyzed gene family expansion and contraction along the phylogenetic tree using CAFE5 (Mendes et al., 2021), which estimates changes in gene family sizes under a stochastic birth‐and‐death model with a single global λ rate across the phylogeny. Significance was assessed using family‐wide *p*‐values as implemented in CAFE5, and gene families with *p* < 0.05 were considered significantly expanded or contracted. The resulting likelihood‐based analyses identified significantly expanded and contracted gene families along specific evolutionary branches.

## 3 RESULTS

### 3.1 Sequencing and genome assembly of *P. calleryana*

High‐quality sequencing data were generated from *P. calleryana* using PacBio HiFi and Hi‐C technologies. A total of 40.18 Gb of PacBio HiFi long reads and 25.70 Gb of clean Hi‐C paired‐end reads were obtained (Table S3). The genome size was estimated to be approximately 454 Mb based on k‐mer analysis, with a heterozygosity rate of 1.4% (Figure 1e). Based on the genome size estimate used for sequencing‐depth calculation, the PacBio HiFi and Hi‐C datasets corresponded to approximately 44.25× and 28.30× coverage, respectively (Table S3).

**FIGURE 2:**
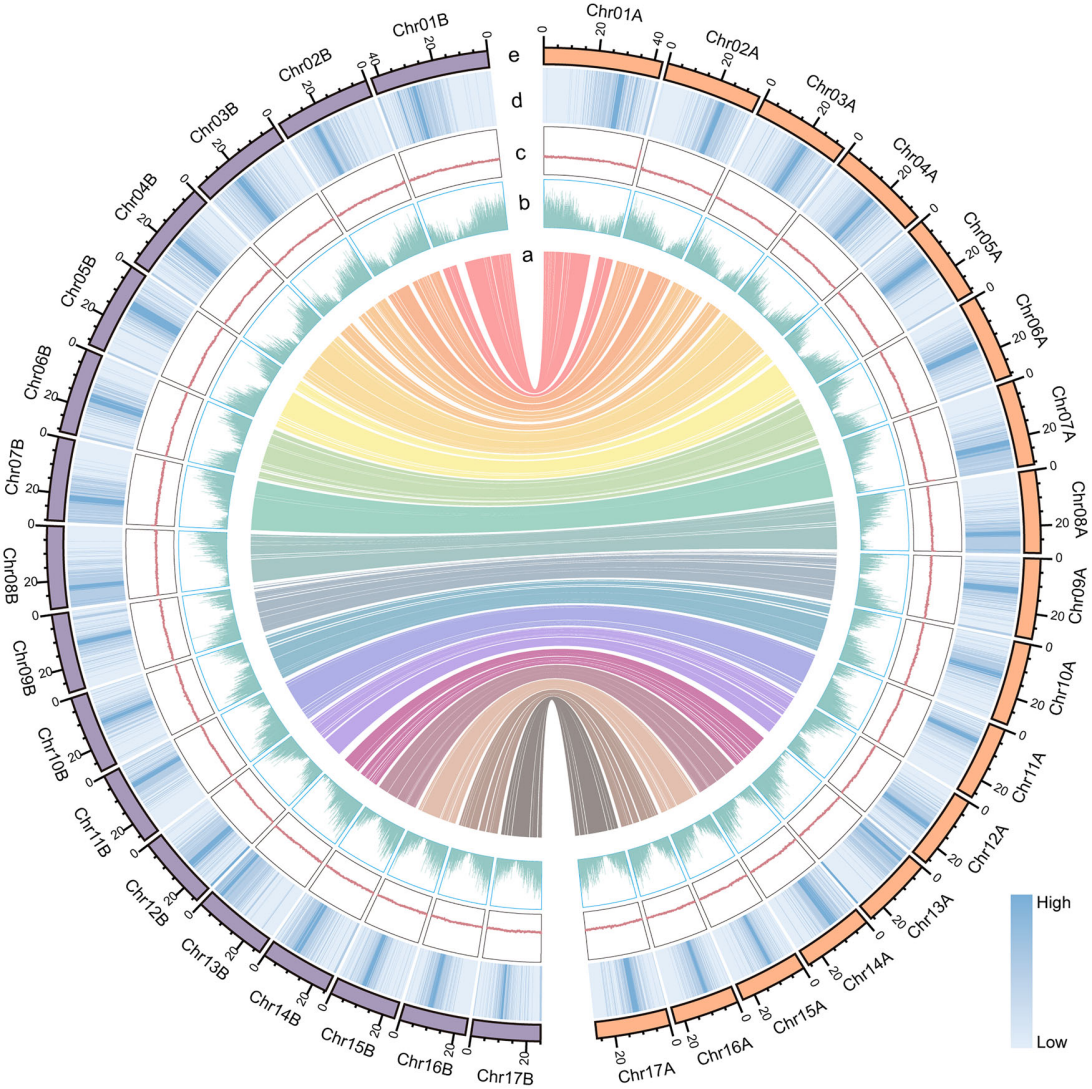
The landscape of genome assembly and annotation of *P. calleryana*. Tracks from inside to outer correspond to (a) inter‐haplotype collinearity between Haplotype A and Haplotype B, (b) gene density (display range 0.00–1.00), (c) GC content (y‐axis range 0.30–0.60), (d) repeat density (y‐axis range 0.00–1.00), and (e) chromosomes of *P. calleryana*. In the heatmap, blue represents a high percentage, while white indicates a low percentage.

Each haploid assembly consisted of 17 chromosomes (Figure 2). For Haplotype A, 18 contigs were assembled with a combined length of 506.97 Mb, while Haplotype B contained 20 contigs totaling 504.62 Mb. Corresponding N50 values reached 29.90 and 28.33 Mb (Table 1). Telomere identification showed that 12 of the Haplotype A chromosomes were capped at both ends, whereas five possessed a telomere on only one side. In Haplotype B, one‐sided telomeres were found on six chromosomes, with the remaining 11 chromosomes displaying telomeric signals at both ends (Figure S1). Additionally, centromeric regions were identified on all 17 chromosomes in both haplotypes using CentIER v2.0 (Figure S1). The k‐mer‐based estimate (∼454 Mb) was lower than the sizes of the two haplotype assemblies (504–506 Mb), consistent with differences between collapsed k‐mer estimates and haplotype‐resolved genome assemblies.

**TABLE 1:** Summary of the *P. calleryana* genome assembly and annotation.

Assembly parameter	Haplotype A	Haplotype B
Genome size (Mb)	506.97	504.62
GC content (%)	37.37	37.34
Contig number	18	20
Contig N50 (Mb)	29.90	28.33
Scaffold number	17	17
Scaffold N50 (Mb)	29.90	29.89
Gap number	1	3
**Annotation index**		
Number of genes	41,234	41,329
Number of CDSs	335,585	335,023
Number of mRNAs	924,163	921,674
Number of exons	378,415	377,318
Mean CDS segment length (bp)	227	227
Mean mRNA length (bp)	3460	3437
Mean number of exons per mRNA (bp)	9.18	9.13
Mean exon length (bp)	290	290
**Evaluation**		
Complete BUSCOs (genome)	99.6%	99.7%
Complete BUSCOs (genes)	99.2%	98.7%
LTR assembly index (LAI)	22.18	22.59
Quality value (QV)	72.18	78.25

BUSCO analysis showed that both haplotype assemblies possess exceptionally high completeness (Table S4). Haplotype A contained 2795 complete BUSCOs (99.6%), including 1392 complete and single‐copy BUSCOs (49.6%) and 1403 complete duplicated BUSCOs (50.0%). Only two BUSCOs were classified as fragmented, and eight BUSCOs were missing. Similarly, Haplotype B contained 2797 complete BUSCOs (99.7%), comprising 1400 complete and single‐copy BUSCOs (49.9%) and 1397 complete duplicated BUSCOs (49.8%). Fragmented and missing BUSCOs were limited to two and six, respectively. High assembly contiguity was supported by LAI scores of 22.18 for Haplotype A and 22.59 for Haplotype B (Table S4). Such elevated duplication is expected given the duplication history of *Pyrus*. A lineage‐specific WGD shared with apple, together with later segmental duplication events, has collectively contributed to the widespread maintenance of duplicated genes (H. Li et al., 2019; M. Sun et al., 2023; T. Zhang, Qiao, et al., 2022). The quality value (QV) scores of the two assemblies were 72.18 and 78.25, respectively, and the completeness reached 99.37%, confirming the overall high quality of the assembled genomes (Table S4). To further validate the assembly quality, we inspected the Hi‐C contact matrices (Figure S2). The pronounced diagonal interaction patterns reflected intact chromatin organization and supported the correctness of the assembly.

### 3.2 Genome annotation

Repeat annotation further characterized a major structural component of the *P. calleryana* genome. TEs occupied nearly half of each haplotype assembly, accounting for 247.61 Mb (48.84%) in Haplotype A and 245.47 Mb (48.64%) in Haplotype B (Table S5). The repeat landscapes were highly similar between the two haplotypes. LTR retrotransposons represented the dominant TE subclass, contributing 35.51% of Haplotype A and 34.08% of Haplotype B. TIR elements and Helitrons accounted for 10.45% and 2.05% of Haplotype A, and 10.82% and 2.94% of Haplotype B, respectively. The comparable TE content and subclass composition between the two haplotypes indicate consistent repeat annotation and provide a genome‐wide overview of the major repetitive components in *P. calleryana*. These proportions are consistent with those reported for other Rosaceae species (Cao et al., 2025; Dong et al., 2020; W. Li et al., 2024; M. Sun et al., 2023).

Across the two haplotypes, 41,234 protein‐coding genes were annotated in Haplotype A, whereas Haplotype B harbored 41,329 such genes (Table 1). Functional annotation indicated broad database coverage, 87.85% of genes matched entries in eggNOG, 45.91% were assigned KEGG pathway information, and 78.15% and 80.66% showed similarity to Swiss‐Prot and TrEMBL proteins, respectively. GO terms were obtained for 92.41% of the genes. Overall, 97.18% of all predicted genes could be assigned functional annotations in at least one database (Table S6). Annotation completeness was further evaluated using BUSCO analysis of the predicted gene sets (Table S7). In Haplotype A, 2772 BUSCOs (99.2%) were classified as complete, including 1524 complete and single‐copy BUSCOs (54.3%) and 1248 complete duplicated BUSCOs (44.5%). Fragmented and missing BUSCOs were limited to 19 (0.7%) and 14 (0.5%), respectively. Similarly, Haplotype B contained 2769 complete BUSCOs (98.7%), of which 1539 (54.9%) were complete and single‐copy BUSCOs and 1230 (43.9%) were complete duplicated BUSCOs. Only 21 BUSCOs (0.7%) were fragmented and 15 (0.5%) were missing. Overall, the high proportion of complete BUSCOs and the low numbers of fragmented and missing BUSCOs indicate that gene models are well supported and that the annotation quality is highly reliable for both haplotypes. To place the *P. calleryana* genome in the context of published *Pyrus* genomes, we compared its major assembly and annotation features with those of representative *Pyrus* species (Table S8). The genome size, annotated gene number, repeat content, and assembly completeness of *P. calleryana* were generally comparable to those reported for other *Pyrus* genomes.

### 3.3 Comparison between haplotype assemblies

We used SyRI to identify the syntenic regions and structural variations between our two newly assembled haplotype genomes. This comparison identified 3,744,017 SNPs and 282 InDels, including 134 insertions and 148 deletions. Additionally, 138 duplications (DUPs) totaling 1.7 Mb, 63 inversions totaling 4.5 Mb, and 330 translocations (TRANSs) totaling 2.9 Mb were detected (Figure S1). Collectively, the detected SNPs and structural variants reveal substantial haplotype divergence in *P. calleryana*. This pattern matches its highly outcrossing mating system and is likely to provide the standing variation that underpins its broad ecological tolerance.

### 3.4 Genome evolution and phylogenetic analysis

**FIGURE 3:**
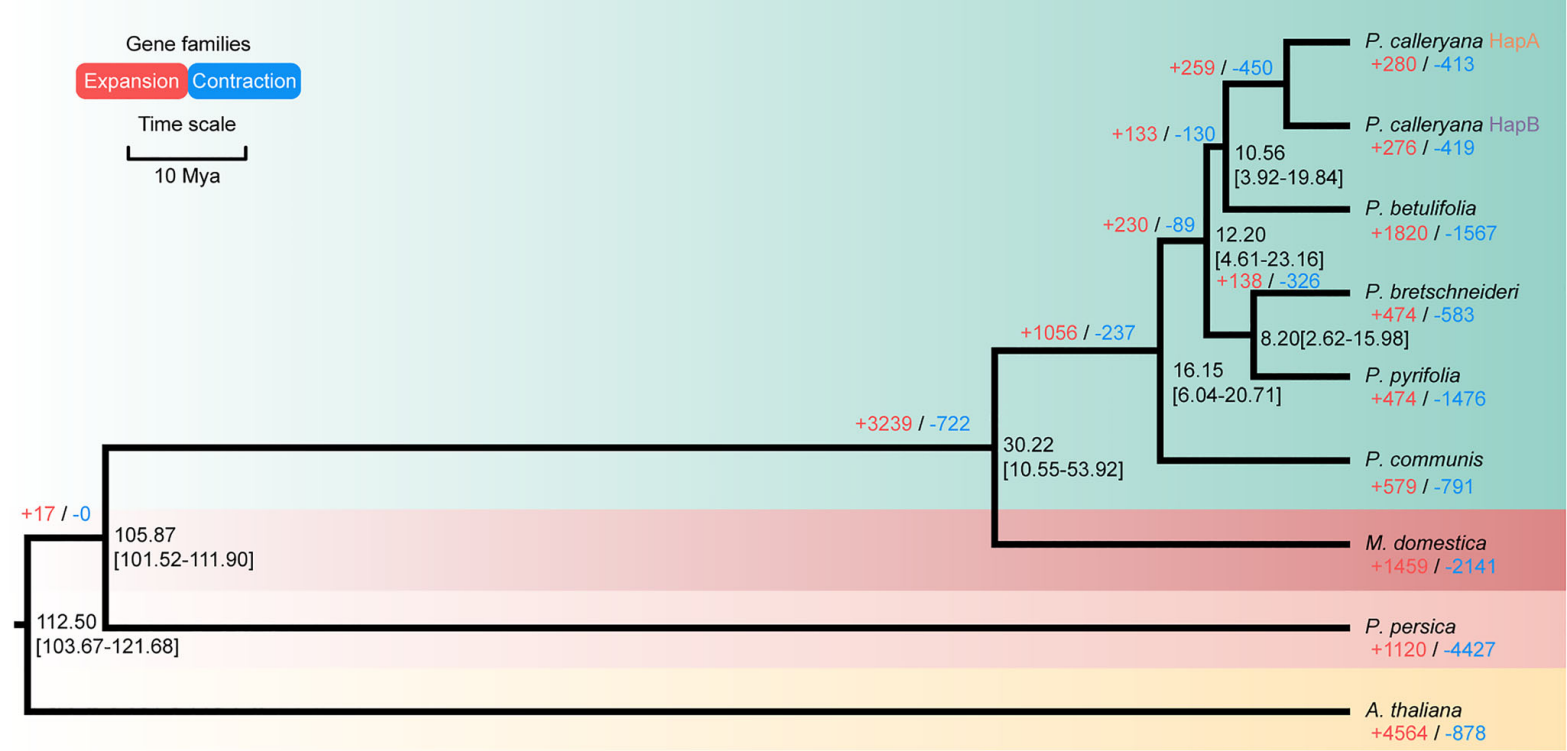
Phylogenetic analysis of *P. calleryana*. The estimated species divergence time is shown on the phylogenetic tree. Gene family expansion (red) and contraction (blue) are illustrated on the branches and nodes of the tree.

We detected a total of 664 single‐copy orthologous genes among eight species (*P. bretschneideri*, *P. calleryana, P. communis*, *P. betulifolia*, *P. pyrifolia*, *M. domestica*, *P. persica*, and *A. thaliana*) using OrthoFinder (Emms & Kelly, 2019). We generated a phylogenetic tree based on concatenated alignments, and the inferred relationships were consistent with previous phylogenetic reconstructions of *Pyrus* evolution (Cao et al., 2025; Dong et al., 2020; Gao et al., 2021). Divergence time estimation indicated that *P. calleryana* separated from *P. betulifolia* approximately 10.56 million years ago (Mya) (Figure 3).

The CAFE5 analysis revealed extensive variation in gene family size across different lineages. *Arabidopsis thaliana* showed the largest number of expanded gene families (+4564), whereas *P. persica* and *M. domestica* exhibited substantial gene family contraction. Within the genus *Pyrus*, *P. betulifolia* displayed the greatest number of expansions (+1820), followed by *P. communis* (+579). Contraction events were also observed, with *P. betulifolia* and *P. pyrifolia* showing notable reductions in gene family size (Figure 3). In *P. calleryana*, we used Haplotype A as a representative, in which 280 gene families were expanded and 413 were contracted (Figure 3). Among these, a total of 1281 genes were identified within expanded gene families. GO enrichment indicated that expanded families were enriched in broad categories including RNA processing and turnover, translation, primary and secondary metabolism, as well as intracellular transport and signaling (Table S9).

**FIGURE 4:**
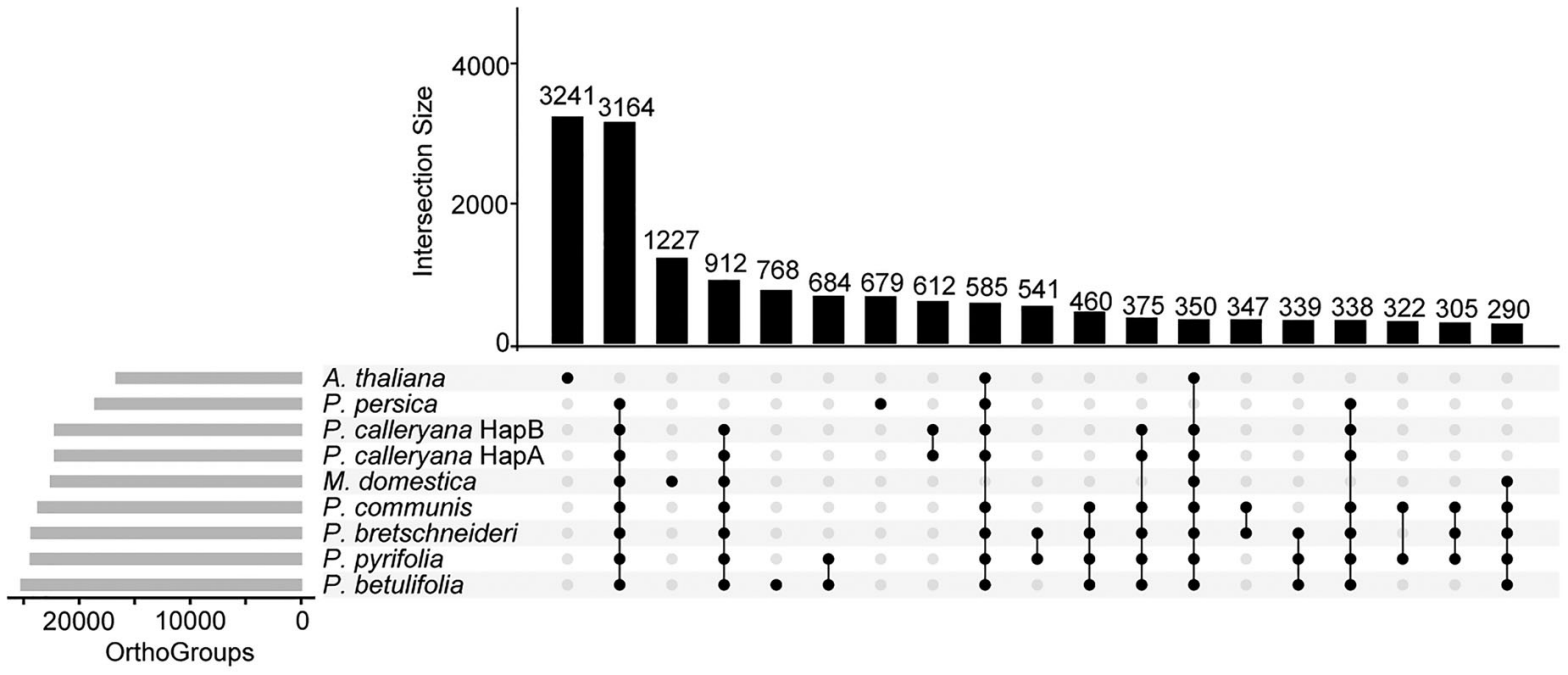
Upset plot of shared and unique orthogroups in the genomes used in phylogenetic analysis.

Across the eight analyzed species, *A. thaliana* showed the highest number of species‐specific orthogroups (3241), followed by *M. domestica* (1227) (Figure 4). A total of 612 orthogroups were shared by *P. calleryana* HapA and HapB, representing orthogroups conserved across both haplotypes (Figure 4). Meanwhile, 680 orthogroups, comprising 1476 genes in total, were present in at least one of the two haplotypes but absent from all other analyzed species, and were therefore defined as *P. calleryana*‐specific orthogroups. To investigate their potential functional roles, we performed GO enrichment analysis on this species‐specific gene set. The enriched terms were mainly associated with hormone‐mediated signaling, amino acid and glycosyl compound metabolism, jasmonic acid and ethylene‐dependent systemic resistance, calcium‐mediated signaling, RNA modification, and cellular responses to endogenous and oxidative stimuli (Table S10). These results suggest that species‐specific orthogroups in *P. calleryana* are preferentially involved in growth regulation (GO:0009742, GO:0009828, GO:0050821, and GO:0001934), metabolic adjustment (GO:1901657, GO:0008652, and GO:0009251), and stress‐responsive processes (GO:0009861, GO:0071495, GO:0034599, and GO:0009626), which may contribute to the ecological adaptability of this species. Notably, several genes in this set were annotated as LIK1‐like candidates based on sequence similarity to *Arabidopsis* LIK1 (AT3G14840). These genes were located on chromosome 4 in both haplotypes and may represent duplicated LIK1‐like LRR‐RLK (where LRR stands for leucine‐rich repeat) candidates associated with stress‐related functions (Table S11).

## 4 DISCUSSION

The *P. calleryana* genome assembled in this study exhibits high contiguity and completeness. Despite the high heterozygosity of this species, we successfully generated a haplotype‐resolved, chromosome‐scale assembly, with each haplotype spanning approximately 505 Mb across 17 chromosomes, matching the established diploid chromosome count (2n = 34) of Callery pear (Nowicki et al., 2022; Sapkota et al., 2022). The resulting genome also shows clear telomeric and centromeric signals on most chromosomes, indicating well‐resolved chromosomal structure (Figure S1). Quality assessment further supports its robustness (Figure S2 and Table S4). We annotated roughly 41,300 protein‐coding genes in each *P. calleryana* haplotype, which is similar to the gene count observed in *P. bretschneideri* (M. Sun et al., 2025). Collectively, these results establish a high‐quality reference genome for Callery pear, providing a solid foundation for downstream genomic analyses of this species.

Our comparative analyses clarify the evolutionary placement of *P. calleryana* and illuminate its genomic history. Phylogenomic reconstruction using 664 single‐copy orthologs resolved *P. calleryana* and *P. betulifolia* as sister lineages, offering a robust framework for interpreting their evolutionary relationships. Divergence time estimates indicate that these two species separated approximately 10.5 Mya, suggesting an early diversification event within the East Asian pear lineage. The two haplotypes exhibit substantial genomic heterogeneity, including abundant SNPs and structural variants. The inversion‐rich region observed on Chr12 further highlights localized structural heterogeneity between the two haplotype assemblies, although its potential functional relevance remains to be evaluated in future population‐level analyses (Figure S1). This deep genomic heterogeneity reflects the high outcrossing rate and long‐term genetic diversity of *P. calleryana*, which may underlie its well‐documented ecological adaptability and invasive potential.

Among the 680 orthogroups that were identified as species‐specific in *P. calleryana*, GO enrichment showed that these genes participate in a broad array of biological processes, including several categories related to stress responses. In particular, we detected significant enrichment of GO:0009861 (jasmonic acid and ethylene‐dependent systemic resistance). Closer inspection of the species‐specific gene sets underlying this GO term revealed three copies of an LRR transmembrane protein kinase on Chr 4 in each haplotype (Table S11). These genes were annotated as LIK1‐like candidates based on similarity to *Arabidopsis* LIK1 (AT3G14840), a receptor‐like kinase involved in chitin‐triggered immunity through its interaction with CERK1. LIK1 acts as a CERK1‐dependent regulator of chitin‐triggered immunity, with *lik1* mutants displaying pathogen‐specific resistance phenotypes that reflect its function in modulating JA/ET‐associated defense signaling (Le et al., 2014). Similar LRR receptor‐like kinases may have been linked to stress‐associated defense responses across plant lineages. In Rosaceae, several LRR‐RLKs, including LRPK in *P. pyrifolia* and multiple members induced during apple scab resistance, have been associated with disease defense (J. Sun et al., 2017). Beyond Rosaceae, genes such as *OsLRR‐RLK1* in rice and *MRK1* in tomato function as positive regulators of herbivore or multi‐stress resistance (Hu et al., 2018; Ma et al., 2022). The presence of several LIK1‐like LRR‐RLKs within the species‐specific orthogroups, along with their known roles in JA/ET‐dependent immune signaling, may indicate lineage‐specific diversification related to stress‐response pathways in *P. calleryana*, although experimental validation will be required to determine their functional significance.

Overall, this high‐quality genome assembly closes a critical gap in *Pyrus* genomics and provides a valuable foundation for future research. It enables in‐depth exploration of the genetic basis of ecological adaptability, invasive capacity, and evolutionary history in *P. calleryana*. Moreover, the haplotype‐resolved structure offers opportunities to investigate allele‐specific variation, structural heterozygosity, and their functional consequences. This genomic resource will facilitate comparative analyses across *Pyrus*, support breeding efforts involving stress‐resilient rootstocks, and inform management strategies for invasive *P. calleryana* populations. Future work integrating population genomics, functional assays, and environmental data will further illuminate the mechanisms driving the success and adaptability of this species.

## 5 CONCLUSIONS

Here, we generated the first chromosome‐scale, haplotype‐resolved genome assembly for *P. calleryana*. Comparative genomic analyses clarified its evolutionary placement within *Pyrus* and identified candidate orthogroups potentially associated with stress responses and environmental adaptation. This genome provides a foundation for future population genomic, functional, and breeding‐related studies of wild pear species.

## AUTHOR CONTRIBUTIONS

**Xiaogang Dai**: Data curation; formal analysis; methodology; software; writing—original draft; writing—review and editing. **Xiaoyue Yang**: Formal analysis; methodology; software; writing—original draft; writing—review and editing. **Changci Jiang**: Data curation; writing—review and editing. **Lizhen Huang**: Data curation; writing—review and editing. **Fangping Li**: Data curation; formal analysis; methodology; writing—review and editing. **Zhuangwei Hou**: Data curation; formal analysis; methodology; writing—review and editing. **Fenghou Shi**: Resources; writing—review and editing. **Zefu Wang**: Funding acquisition; methodology; supervision. **Wenjie Yang**: Conceptualization; formal analysis; methodology; resources; software; supervision; writing—original draft; writing—review and editing. **Yongbao Shen**: Conceptualization; funding acquisition; methodology; project administration; resources; supervision; writing—original draft; writing—review and editing.

## CONFLICT OF INTEREST STATEMENT

The authors declare no conflicts of interest.

## Supporting information




**Table S1**. Summary of RNA‐seq datasets downloaded from NCBI for genome annotation.
**Table S2**. Data source of genome dataset used in phylogenetic analysis.
**Table S3**. Statistics of genomic sequencing data.
**Table S4**. Evaluation of *P. calleryana* genome assembly.
**Table S5**. Summary of the *P. calleryana* genome repeat annotation.
**Table S6**. Summary of the *P. calleryana* genome gene functional annotation.
**Table S7**. BUSCO assessment of predicted genes in the *P. calleryana* genome.
**Table S8**. Comparison of genome assembly and annotation features between *P. calleryana* and published *Pyrus* genomes.
**Table S9**. GO enrichment of genes in expanded gene families of *P. calleryana* genome.
**Table S10**. GO enrichment of genes from species‐specific orthogroups in the *P. calleryana* genome.
**Table S11**. Functional annotation of genes from species‐specific orthogroups in the *P. calleryana* genome.

**Figure S1**. Structural variation between the two haploid genomes of *P. calleryana*.
**Figure S2**. Inter‐chromosomal Hi‐C heatmap of *P. calleryana* genome. The heatmap displays chromatin interaction frequencies across all chromosomes, derived from Hi‐C sequencing data and normalized using the Knight–Ruiz (KR) algorithm. The color scale indicates the intensity of chromatin contacts.

## Data Availability

The raw sequence data reported in this paper have been deposited in National Genomics Data Center, Beijing Institute of Genomics, Chinese Academy of Sciences, and China National Center for Bioinformation, under the project identifier PRJCA047760. The assembled genome files are also deposited in the Genome Warehouse in National Genomics Data Center under accession numbers GWHGQOO00000000.1 and GWHGQOP00000000.1. The assembly, annotation, and variant records between the two haplotypes can be downloaded at https://doi.org/10.6084/m9.figshare.30334567.v2.
