# Supplementary material for: A high‐quality haplotype‐resolved genome of Pyrus calleryana provides insights into adaptive gene families
Source: Plant Genome. 2026 Jul 29;19(3):e70286. doi: 10.1002/tpg2.70286 (PMC13431685; doi:10.1002/tpg2.70286)
Supplement: Supplementary file 1 [file TPG2-19-e70286-s001.docx]

**Figure S1. Structural variation between the two haploid genomes of *P. calleryana*.**

**Figure S2. Inter-chromosomal Hi-C heatmap of *P. calleryana* genome.** The heatmap displays chromatin interaction frequencies across all chromosomes, derived from Hi-C sequencing data and normalized using the Knight–Ruiz (KR) algorithm. The color scale indicates the intensity of chromatin contacts.
